# Favorable Alleles for Stem Water-Soluble Carbohydrates Identified by Association Analysis Contribute to Grain Weight under Drought Stress Conditions in Wheat

**DOI:** 10.1371/journal.pone.0119438

**Published:** 2015-03-13

**Authors:** Weiyu Li, Bin Zhang, Runzhi Li, Xiaoping Chang, Ruilian Jing

**Affiliations:** 1 National Key Facility for Crop Gene Resources and Genetic Improvement / Institute of Crop Science, Chinese Academy of Agricultural Sciences, Beijing 100081, China; 2 Agronomy College, Shanxi Agricultural University, Taigu 030801, China; Huazhong University of Science & Technology(HUST), CHINA

## Abstract

Drought is a major environmental constraint to crop distribution and productivity. Stem water-soluble carbohydrates (WSC) buffer wheat grain yield against conditions unfavorable for photosynthesis during the grain filling stage. In this study, 262 winter wheat accessions and 209 genome-wide SSR markers were collected and used to undertake association analysis based on a mixed linear model (MLM). The WSC in different internodes at three growth stages and 1000-grain weight (TGW) were investigated under four environmental regimes (well-watered, drought stress during the whole growth period, and two levels of terminal drought stress imposed by chemical desiccation under the well-watered and drought stress during the whole growth period conditions). Under diverse drought stress conditions, WSC in lower internodes showed significant positive correlations with TGW, especially at the flowering stage under well-watered conditions and at grain filling under drought stress. Sixteen novel WSC-favorable alleles were identified, and five of them contributed to significantly higher TGW. In addition, pyramiding WSC favorable alleles was not only effective for obtaining accessions with higher WSC, but also for enhancing TGW under different water regimes. During the past fifty years of wheat breeding, WSC was selected incidentally. The average number of favorable WSC alleles increased from 1.13 in the pre-1960 period to 4.41 in the post-2000 period. The results indicate a high potential for using marker-assisted selection to pyramid WSC favorable alleles in improving WSC and TGW in wheat.

## Introduction

Drought, defined as water deficit, is one of the major environmental factors determining crop distribution and productivity. Plants exposed to drought undergo dramatic losses in productivity if they are not adapted to cope with such conditions [1]. Wheat (*Triticum aestivum* L.) is one of the most important crops in the world and any loss of yield has serious consequences, both locally and worldwide. For dryland wheat grown in arid and semiarid areas, such as the semi-arid regions of northwest China and the Mediterranean region, low and erratic rainfall can greatly reduce grain yield and yield stability [2]. Terminal drought stress from unrelieved water deficit that may occur during and after flowering impairs photosynthesis, enhances plant senescence and influences the duration of grain filling [3]. Understanding plant tolerance to drought is therefore of fundamental importance and forms one of the major research topics in agronomy [1].

It was suggested that yield should be taken as an important index of crop drought resistance [4,5]. Water-soluble carbohydrates (WSC; composed mainly of fructans, sucrose, glucose, and fructose, with the main reserve as fructans at the late stages of WSC accumulation) deposited in wheat stems are important carbon sources for grain filling [6]. Moreover, fructans can act as compatible solutes in cells under osmotic stress [7]. Stem water-soluble carbohydrates (WSC) can be an important contributor to buffer grain yields against unfavorable conditions for photosynthesis during grain-filling period [8]. Mobilization of WSC during grain filling can potentially contribute about 20% of the final grain weight under non-stress conditions, and up to 70% or more of grain dry matter under drought stress in wheat [7,9]. The amount of WSC accumulation and remobilization reportedly differs between internodes [10,11].

WSC, which mainly occurs as fructans, increased in response to water deficit, and WSC were higher in drought tolerant genotypes than those in sensitive ones [7,12]. Grains of sensitive cultivars reached maturity much earlier than tolerant ones under both control and stress conditions [13]. Therefore, it may be concluded that drought tolerant wheat cultivars have a high capability of WSC accumulation, coupled with higher mobilization efficiency, stronger sink activity and longer duration of grain filling. The ability to store and remobilize large amounts of WSC to grain has been suggested as a selection criterion for wheat breeding due to its high heritability and positive linear relationship with grain yield [6,13–15]. However, unpredictable water deficits from year to year make reliable selection difficult in dryland breeding programs. Nicolas and Turner [16] developed a technique, involving use of a leaf spray of potassium iodide as a mild treatment on wheat for revealing genotypic differences in the absence of photosynthesis under terminal stress (post-anthesis stress) in wheat. Blum [9] and Regan et al. [17] found a highly significant relationship between the rates of grain weight reduction caused by chemical desiccation and by drought stress. In wheat breeding, chemical desiccation can be used to assess advanced lines or used in mass selection.

Studies on WSC QTL have been reported in rice [18,19], wheat [12,20], maize [21], barley [22] and perennial ryegrass [23]. QTL studies using three wheat mapping populations showed that WSC accumulation was controlled by many genes, and plays an important role in assuring stable yield and grain size [20]. With the rapid increases in number of molecular markers, association analysis has become an important tool for dissection of complex traits [24]. It makes full use of existing diversity and provides a high-resolution platform for mapping QTL [25]. In wheat, loci influencing various traits, such as stem rust resistance, plant height and grain weight, were identified by association analysis [26–28].

A few loci for WSC were reported through association analysis. In previous research on WSC in genetic populations most attention was given to whole stem WSC. However, such work did not provide sufficient information on the genetics of WSC, because 1) WSC in different internodes responds differently to drought; and 2) family-based genetic populations possess limited favorable alleles because they are derived from two parents. In the present study, a diverse population of 262 winter wheat accessions was tested with 209 SSR markers distributed on all 21 chromosomes by association analysis aimed at gaining further genetic insight into the genetic mechanism of WSC under various drought treatments. We investigated WSC in different internodes at three growth stages and 1000-grain weight (TGW) at two growth stages in four environments (well-watered, drought stress during the whole growth period, and two levels of terminal drought stress imposed by chemical desiccation under the well-watered and drought stress during the whole growth period conditions). Our objectives were to: 1) further describe the effect of various drought conditions on WSC in different internodes; 2) assess the relationship between WSC and TGW at three growth stages; 3) identify elite alleles significantly associated with WSC under various drought conditions; and 4) confirm the close genetic relationships between WSC and TGW.

## Materials and Methods

### Ethics Statement

Two locations, Changping (116°13´E; 40°13´N) and Shunyi (116°56´E; 40°23´N) in Beijing, are the experiment stations of the Institute of Crop Science, Chinese Academy of Agricultural Sciences. We have obtained the relevant permission for our field studies for growing our plant materials in the field from the corresponding institutions. There was no specific permissions required for these locations/activities. Our field studies did not involve endangered or protected species.

### Plant materials and field experiments

A total of 262 common wheat accessions collected as a diverse population for this research (S1 Table). Of them, 254 were from China, 3 from USA, 2 from Australia, 2 from Italy, and 1 from Romania. The cultivars from China were mainly planted in the Northern Winter Wheat Zone, and Yellow and Huai River Valleys Facultative Wheat Zone [29]. The population was sown in Changping, Beijing (116°13´E; 40°13´N), at the beginning of October 2010 and harvested in mid-June 2011. The experimental unit was a 2 m four-row plot, with 30 cm between the rows. Forty seeds were planted per row. The field was managed under separate rain-fed (drought stress, CK-DS) and well-watered (CK-WW) conditions. The rainfall from sowing to harvest was 131 mm. The WW treatment was watered with 750 m^3^/ha (75 mm) at the pre-overwintering, booting, flowering and grain filling stages, respectively. The 0.8% chemical desiccant KI (Potassium Iodide, AR, Sinopharm Chemical Reagent Co., Ltd) was sprayed on the leaves of each plot separately at the third day after flowering to simulate the terminal drought stress [17,30–32]. There were four water regimes including well-watered as a control (CK-WW), drought stress during the whole growth period (CK-DS) and two levels of terminal drought stress imposed under the well-watered and drought stress during the whole growth period conditions (KI-WW; KI-DS).

### Phenotyping of WSC and TGW

Methods of collecting data on stem water-soluble carbohydrates (WSC)-related and TGW were reported earlier [12,33]. For each accession under the four water regimes, five main stems with the same heading date were selected as samples. The main stem was cut from the soil surface at three morphological stages, viz., flowering, mid-grain filling (14 days after flowering, DAF), and maturity. Leaf blades were removed, and the stems with leaf sheaths were cut into three parts, the upmost internode (peduncle, Ped), the lower internode (the remainder segments of stem except for peduncle, Low) and the spike. Stem samples for each accession were chipped into 2–5 mm lengths. The WSC of the three sections, i.e. peduncle, the lower internode and whole stem (Ste), were determined by different near-infrared reflectance spectroscopy (NIRS) regression models, which were developed for quantitative determination of WSC using modeling samples of 150 DH (Hanxuan 10 × Lumai 14) lines [33]. Briefly, at the first step, partial least square regression models for predicting WSC in the target parts of wheat were developed using selected wavelength regions, spectroscopic pretreatments and the latent variables included in each model. The total amounts of WSC (mg WSC / g dry weight, mg / g dw) in each modeling sample of 150 DH lines were also measured by chemical assay (anthrone colorimetric assay), and used for the cross validation. The NIRS regression models were highly accurate in determination of the true values of WSC measured by chemical assay in the wheat organs tested, according to high coefficients of determination (R^2^ >0.992) and low root mean square errors of prediction (RMSEP <0.228). An additional 40 samples per model (i.e. not included in the modeling samples) were used to verify the accuracy of outer predictions of models. Paired *t*-tests were also conducted using the outer samples to assess the models. The results showed the high accuracy of the models in predicting WSC. We obtained WSC at the flowering (WSCF), mid-grain filling (WSCG), and maturity (WSCM) stages.

Spikes corresponding to main stem samples were collected at the mid-grain filling and maturity stages for each accession to obtain 1000-grain weight (TGWG, TGWM). The grain-filling efficiency (GFE, %) at an early period (before 14 DAF) and a late period (from 14 DAF prior to maturity) were assessed by [TGWG / TGWM] × 100% and [(TGWM − TGWG) / TGWM] × 100%, respectively [12].

### SSR genotyping and association mapping

Two hundred and nine SSR markers, evenly spaced along the 21 wheat chromosomes, were selected for evaluating population structure, relative kinship, and association mapping. The genetic positions of these SSR markers were from the consensus map Ta-SSR-2004 [34] and the Komugi wheat genetic resources database (http://www.shigen.nig.ac.jp/wheat/komugi​/top/top.jsp). Molecular data were obtained by a fluorescence detection system [35]. Amplification products were separated on an ABI3730 DNA Analyzer, and fragment sizes were calculated by GeneMapper software (Applied Biosystems).

Allele number, allele frequency and polymorphism information content (PIC) were calculated by PowerMarker V3.25 (Lui and Muse, 2005). Population structure was estimated by STRUCTURE v2.3.2 using data from 209 SSR markers. Ten subpopulations (*k* = 1 to 10) were set with a burn-in period of 50,000 iterations and a run of 500,000 replications of Markov Chain Monte Carlo after burn in. The △*k* method was applied according to LnP(D) in the STRUCTURE, and the output and result were estimated [36]. The *Q* data of five replicate runs were integrated by CLUMPP software [37]. Principal coordinate analysis based on genetic distances was also used to confirm the results of STRUCTURE by NTSYSpc analysis [38,39]. The relative kinship coefficient (*K*) was calculated by the SPAGeDi software package [40]. Finally, the *Q* + *K* models were performed using mixed linear model (MLM) in tassel V2.1 for association of WSC and TGW [24].

## Results

### Trait variation

**Fig 1:**
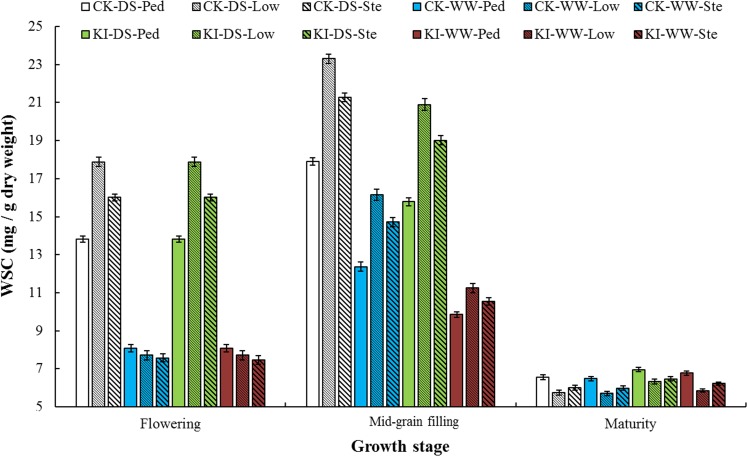
Stem water-soluble carbohydrates (WSC, mg / g dw) of 262 winter wheat accessions in different internodes at three growth stages under well-watered and drought stress environments. Bars indicate 2×SE. CK-DS-Ped, peduncle under drought stress; CK-DS-Low, lower internode, drought stressed; CK-DS-Ste, whole stem, drought stressed; CK-WW-Ped, peduncle, well-watered; CK-WW-Low, lower internode, well-watered; CK-WW-Ste, whole stem, well-watered; KI-DS-Ped, peduncle treated with KI, drought stressed; KI-DS-Low, lower internode treated with KI, drought stressed; KI-DS-Ste, whole stem treated with KI, drought stressed; KI-WW-Ped, peduncle treated with KI, well-watered; KI-WW-Low, lower internode treated with KI, well-watered; KI-WW-Ste, whole stem treated with KI, well-watered.

**Fig 2:**
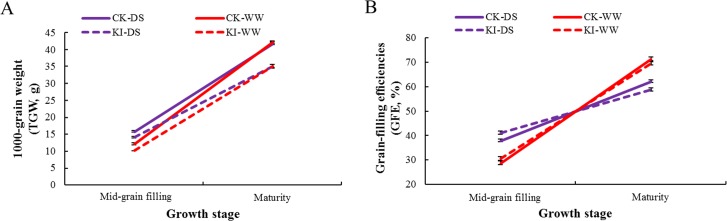
1000-grain weight (TGW, a) and grain-filling efficiency (GFE, b) based on 262 winter wheat accessions. Bars indicate 2×SE.

A total of 262 common wheat accessions with similar flowering time were collected as a diverse population for our research. The average values of 262 wheat accessions for WSC, TGW and grain-filling efficiency (GFE) were presented in S2 and S3 Tables, Fig. 1 and Fig. 2. There was considerable phenotypic variability for WSC and TGW in the natural population with coefficients of variation (*CV*) ranging from 16.11 to 50.51% and from 14.65 to 43.64%, respectively. The WSC at the mid-grain filling were higher than those at flowering and maturity stages. Before maturity, the WSC was overall much higher in lower internodes than that in the peduncles in every water regime (Fig. 1; S2 Table).

### Correlations between WSC and TGW

Pearson correlation coefficients were calculated to further understand the relationships between WSC of internodes and TGW under diverse water regimes. There were significant correlations between WSC and TGW at flowering and mid-grain filling stages. Compared to WSC in the peduncle, a higher correlation existed between WSC in lower internodes and TGW (data not presented). Under diverse drought stress, WSC of lower internodes at the mid-grain filling (WSCG) showed significant correlations with TGW at the mid-grain filling (r = 0.313*** and 0.302*** under CK-DS and KI-DS) and those at maturity (0.358***, 0.382*** and 0.257** under CK-DS, KI-DS and KI-WW) (Table 1). However, under well-watered condition (CK-WW), a significant positive correlation (r = 0.197**) between WSC of the lower internode and TGW at maturity was detected at the flowering stage, but there were no significant correlations at other growth stages (Table 1).

**Table 1:** Pearson correlation coefficients of WSC of lower internodes and TGW under various water conditions.

WSC	1000-grain weight (TGW)			
		CK-DS	CK-WW	KI-DS	KI-WW
WSCF	0.249***/0.216***	0.058/0.197**	0.113/0.157*	0.105/0.028
WSCG	0.313***/0.358***	0.045/0.085	0.302***/0.382***	0.127/0.257**
WSCM	0.088/0.054	0.010/0.057	-0.126*/-0.157*	-0.034/0.065

* Significant at *P* = 0.05

** Significant at *P* = 0.01

*** Significant at *P* = 0.001. Numbers at the left of the “/” were 1000-grain weight measured at the mid-grain filling and those at the right were measured at maturity. WSCF, stem water-soluble carbohydrates at flowering; WSCG, stem water-soluble carbohydrates at the mid-grain-filling; WSCM, stem water-soluble carbohydrates at maturity; CK-DS, drought stress condition; CK-WW, well-watered condition; KI-DS, treated with KI (potassium iodide) under drought stress condition; KI-WW, treated with KI under well-watered condition.

### Allelic diversity and population structure

**Fig 3:**
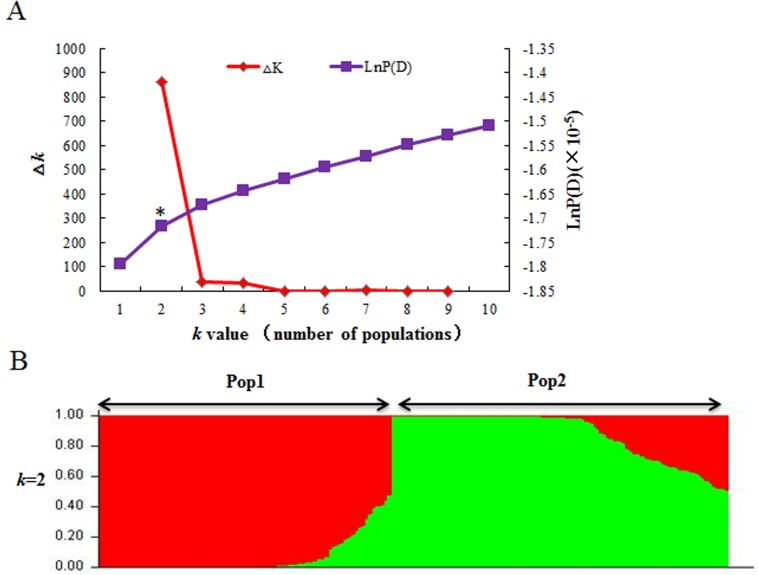
Population structure analysis of 262 wheat accessions based on 209 SSR markers. (A) Estimated LnP(D) and △*k* over five repeats of STRUCTURE analysis; (B) Two sub-populations inferred by structure analysis. Each of the 262 individuals is represented by a vertical line and different colors indicate different sub-populations. Asterisk in (A) indicates the point of the most significant change in the LnP(D) value.

**Fig 4:**
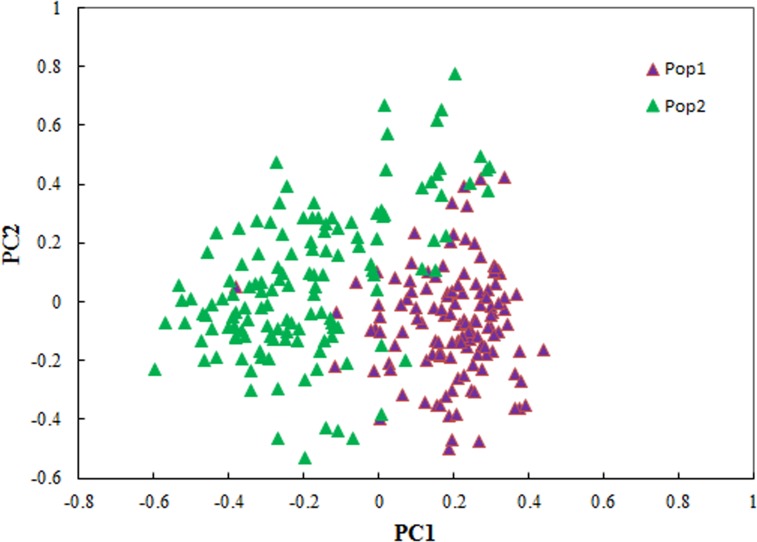
Principal coordinate analysis of 262 wheat accessions based on 209 SSR markers reveals two sub-populations.

A total of 2,748 alleles were identified at the 209 SSR loci in our population (262 accessions), an average of 13/locus. The average percentage of missing data for all markers and genotypes was 3.36%. The polymorphism information content (PIC) of the SSR loci ranged from 0.0175 to 0.932 with an average of 0.619. Population structure was assessed by STRUCTURE v2.3.2 using all markers. The most significant change of the LnP(D) value was observed at *k* = 2, and there was also a sharp peak of △*k*, indicating that *k* = 2 was the best separator (Fig. 3). This was further supported by principal coordinate analysis based on Nei’s genetic distances [38]. The top two principal components clearly separated the 262 wheat accessions into two sub-populations, comprising 126 and 136 accessions, respectively, and agreeing the with the STRUCTURE analysis (Fig. 4).

### Association studies for WSC and TGW

According to the correlation results, WSC of the lower internode (WSCF under CK-WW, WSCG under CK-DS, KI-WW and KI-DS) and TGW at maturity under four water regimes were used for association mapping. Moreover, WSC of the whole stem were also included to further identify associated loci. A mixed linear model (MLM) was used; it accounted for population structure (*Q*) and unequal relatedness among individuals (*K* matrix) [41]. Thirty-three novel loci were significantly (*P* <0.01) associated with WSC (S4 Table). Sixteen loci were significantly associated with TGW at maturity (S5 Table). In regard to WSC, *Xgwm186–5A* was detected under both KI-WW and KI-DS; *Xgwm413–1B*, *Xbarc324–3A*, *Xgwm566–3B* and *Xbarc175–6D* were associated with both WSC in lower internodes and in the whole stems under KI-DS, CK-DS, CK-DS and CK-WW, respectively (S4 Table). *Xbarc24–6B*, near QTL *QWSCm*.*cgb-6B*.*1* (flanking marker region: *Xgwm219*–*Xwmc341*) controlling WSC was also detected in a DH population [12]. In Ta-SSR-2004 [34], the genetic distance between *Xbarc24–6B* and *Xgwm219–6B* is 4 cM. *Xbarc324–3A* and *Xgwm361–6B* were simultaneously associated with both WSC and TGW under CK-DS and KI-DS, respectively. These results provided further genetic insight into the significant correlations between WSC and TGW.

### Favorable WSC alleles enhance TGW under diverse water conditions

For each locus associated with WSC, differences in WSC between accessions with the favorable allele and those carrying other alleles were estimated by ANOVA (SAS 8.01). Sixteen of the 33 associated loci had significant favorable allelic effect; that is, the difference between the favorable allele and others was significant (*P* <0.05). There were 4, 3, 7 and 2 favorable alleles under CK-DS, CK-WW, KI-DS and KI-WW, respectively (Table 2). *Xgwm566–3B*_*124*_ was simultaneously associated with both WSCG-Low and WSCG-Ste under CK-DS. Moreover, the WSC of accessions combining larger numbers of favorable alleles were higher than those with lower numbers of favorable alleles, showing that the accumulation of favorable alleles leads to better phenotypes (Table 3).

**Table 2:** Phenotypic values of favorable marker alleles significantly associated with WSC.

Water regime	Trait	Locus	Favorable allele (bp)	Freq. (%)	Mean ± SE (mg / g dw)	*P* value
CK-DS	WSCG-Low	*Xgwm403–1B*	138	5.76	277.3 ± 9.6	0***
			Others	94.24	230.3 ± 2.4	
		*Xgwm566–3B*	124	7.82	256.4 ± 7.2	0.0045**
			Others	92.18	231.1 ± 2.5	
	WSCG-Ste	*Xbarc181–1B*	187	26.75	223.6 ± 4.1	0.0027**
			Others	73.25	208.6 ± 4.3	
		*Xgwm566–3B*	124	7.82	233.2 ± 7.0	0.0069**
			Others	92.18	210.8 ± 2.3	
		*Xgwm537–7B*	207	5.76	231.9 ± 8.5	0.0321*
			Others	94.24	211.4 ± 2.3	
CK-WW	WSCF-Low	*Xgwm358–5D*	162	29.72	91.7 ± 5.2	0***
			Others	70.28	70.9 ± 2.6	
		*Xgwm131–7B*	111 and 118	9.64	103.5 ± 8.9	0.0004***
			Others	90.36	74.3 ± 2.5	
	WSCF-Ste	*Xgwm148–2B*	165	24.02	90.9 ± 4.6	0***
			Others	75.98	70.9 ± 2.5	
KI-DS	WSCG-Low	*Xgwm259–1B*	102	12.3	225.4 ± 8.8	0.046*
			Others	87.7	206.6 ± 3.3	
		*Xgwm261–2D*	203	6.35	232.3 ± 7.8	0.0493*
			Others	93.65	207.3 ± 3.3	
		*Xbarc314–3A*	257	59.13	221.1 ± 3.4	0***
			Others	40.87	191.2 ± 5.3	
		*Xcfd23–4D*	220	61.51	214.2 ± 3.5	0.0288*
			Others	38.49	200.3 ± 5.8	
	WSCG-Ste	*Xgwm374–2B*	217	8.91	209.6 ± 6.4	0.0138*
			Others	91.09	188.2 ± 2.6	
		*Xgwm149–4B*	153	59.92	197.8 ± 2.8	0.0001***
			Others	40.81	178.7 ± 4.3	
		*Xbarc216–5B*	87	20.24	204.9 ± 4.7	0.0024**
			Others	79.76	186.4 ± 2.8	
KI-WW	WSCG-Low	*Xbarc176–7B*	282	10.67	137.7 ± 9.6	0.0003***
			Others	89.33	109.5 ± 2.4	
	WSCF-Ste	*Xgwm186–5A*	130	7.52	124.6 ± 8.6	0.0042**
		* *	Others	92.48	103.9 ± 3.4	

*P* values are for comparison between phenotypic values of favorable allele and others.

*, **, *** indicate significance at *P* = 0.05, 0.01 and 0.001, respectively. Underlined values indicate that lines with the single favorable WSC allele produced significantly (*P* < 0.05) higher TGW at maturity than accessions with other alleles.

**Table 3:** Pyramiding of favorable WSC alleles enhanced WSC and TGW under various water conditions.

Water condition	Favorable allele	No. of alleles	Frequency (%)	WSC Mean ± SE (mg / g dw)	TGW Mean ± SE (g)
CK-DS	4	≥2	12.98	258.0±6.4(a)^1^	43.75±1.02(a) ^1^
		1	22.90	247.0±4.5(a)	42.17±0.72(ab)
		0	64.12	222.6±2.8(b)	41.21±0.49(b)
CK-WW	3	≥2	13.7	108.6±6.2(a)	44.28±1.01(a)
		1	33.2	78.2±4.5(b)	43.62±0.62(a)
		0	53.1	68.2±3.0(b)	40.73±0.53(b)
KI-DS	7	≥3	41.2	226.7±3.8(a)	36.00±0.57(a)
		2	30.9	209.1±5.0(b)	35.46±0.93(ab)
		≤1	27.9	181.6±6.8(c)	33.61±0.91(b)
KI-WW	2	≥1	16.4	134.6±7.4(a)	34.54±1.24(a)
		0	93.6	108.0±2.4(b)	35.15±0.60(a)

^1^ Values with different letters in columns are significantly different (P <0.05).

**Fig 5:**
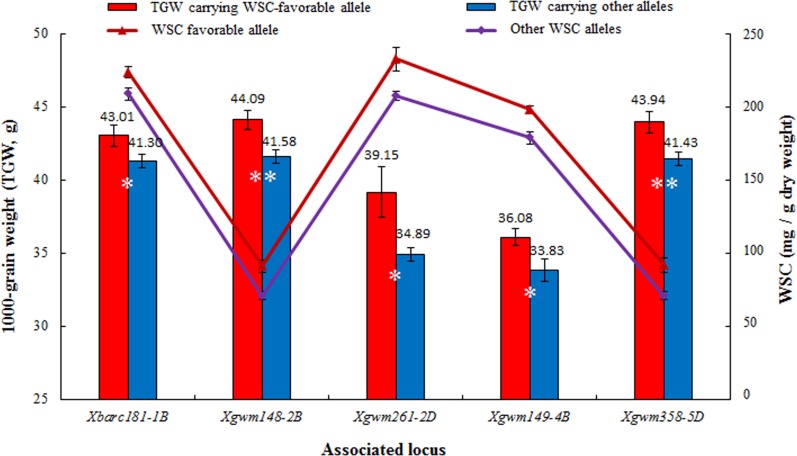
Single WSC favorable allele contributed to significantly higher 1000-grain weight (TGW). Bars indicate 2×SE. WSC corresponding to *Xbarc181–1B*, *Xgwm261–2D* and *Xgwm149–4B* represent WSC at the mid-grain filling under CK-DS, KI-DS and KI-DS, respectively; WSC corresponding to *Xgwm148–2B* and *Xgwm358–5D* represent WSC at flowering under CK-WW. TGW was measured at maturity. *, ** indicate significance at *P* = 0.05 and *P* = 0.01, respectively.

In accordance with the significant positive correlations between WSC and TGW, the TGW of accessions carrying favorable WSC alleles were overall much higher than those of others. Only five of the 16 favorable WSC alleles, individually contributed to significantly higher TGW; they were *Xbarc181–1B*_*187*_, *Xgwm148–2B*_*165*_, *Xgwm261–2D*_*203*_, *Xgwm149–4B*_*153*_ and *Xgwm358–5D*_*162*_ (Fig. 5). We also analyzed the TGW of accessions containing different numbers of favorable WSC alleles under diverse water conditions (CK-WW, CK-DS, KI-WW, KI-DS, Table 3). Under CK-DS, accessions combining more than two favorable WSC alleles had significantly (*P* <0.05) higher TGW (43.75 g) than those without favorable alleles (41.21 g). Except for KI-WW, the pyramiding of favorable WSC alleles was also effective under CK-WW and KI-DS. There were only 4 accessions carrying 2 favorable alleles where significant differences under KI-WW were not detected.

### WSC was selected with TGW during the past fifty years of wheat breeding

**Fig 6:**
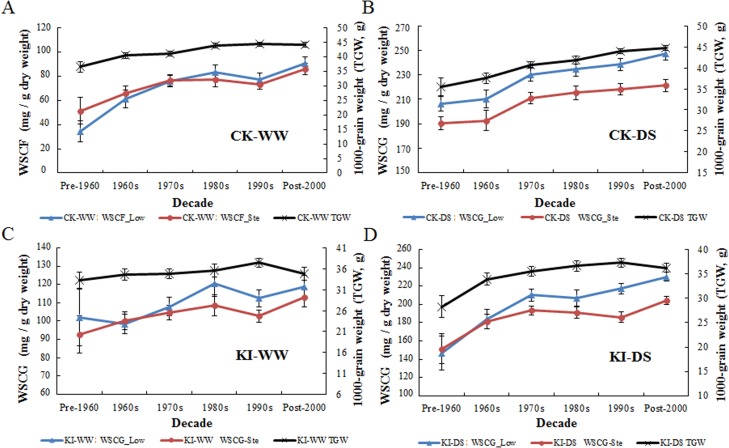
TGW and WSC of modern varieties from different decades under four water regimes. Pre-1960, before 1960; Post-2000, after 2000; There were 8, 27, 54, 39, 58 and 51 accessions released in Pre-1960, during the 1960s, 1970s, 1980s, 1990s, and Post-2000, respectively. Ten landraces and 15 accessions with unknown released decades were excluded. Bars indicate 2×SE.

WSC in lower internodes increased from means of 34.3, 206.6, 101.9, 146.6 before 1960 (pre-1960) to 90.7, 247.2, 118.9, 230.1 after 2000 (post-2000) under CK-WW, CK-DS, KI-WW, KI-DS, respectively; the trend for WSC of whole stems was the same (Fig. 6; S6 Table). Overall, the TGW and WSC of modern varieties gradually increased from pre-1960 to post-2000 under all four water regimes (Fig. 6).

**Fig 7:**
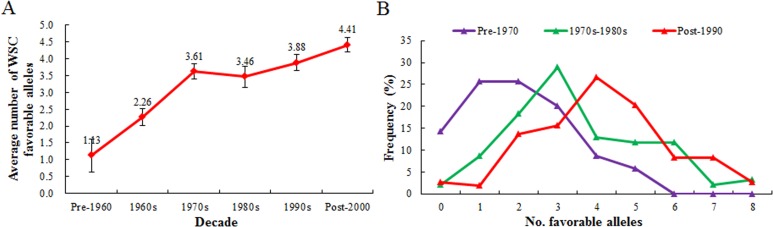
Accumulation (A) and frequency distribution (B) of 16 favorable alleles in modern varieties from different decades. Bars indicate 2×SE.

Breeding leaves a strong footprint at the genome level [42]. A number of important genes (alleles) were positively selected, as implicated by changes in numbers and frequency [43]. We estimated the accumulation and frequency distribution of 16 favorable alleles identified in our research in modern varieties from different decades. The average number of favorable WSC alleles increased from 1.13 in the pre-1960 period to 4.41 in the post-2000 period (Fig. 7A). Most modern varieties carried one or two favorable alleles before 1970, whereas modern varieties after 1990 have as many as eight favorable alleles (Fig. 7B). The obvious positive selection of the 16 favorable alleles identified here proves their value in breeding programs over past decades. However, compared with the 16 favorable alleles, the average number (4.41) in post-2000 varieties is relatively lower and indicates considerable potential for further improvement.

## Discussion

### Drought stress induces higher remobilization efficiency of stem water-soluble carbohydrates

Extensive studies have demonstrated that remobilization of stem water-soluble carbohydrates to grain in wheat require initiation of whole-plant senescence [44]. While drought stress induces early senescence by increasing abscisic acid (ABA) and reducing cytokinins in wheat and rice, it also leads to more and faster remobilization of stem water-soluble carbohydrates from vegetative tissues to the grains and increases the grain filling rate, but shortens the grain filling period [45,46]. In this study, 1000-grain weight and grain-filling efficiency under drought stress conditions was clearly higher compared to those under well-watered condition during the early grain filling period (Fig. 2). However, the remobilization efficiency of stem water-soluble carbohydrates was not sufficient to compensate for the reduction in grain filling period during most kinds of drought conditions [30]. The final values of TGW and grain-filling efficiency in our study were still lower under drought stress conditions than those under well-watered conditions (Fig. 2). However, 1000-grain weight at maturity under well-watered condition is only slightly higher than that those under drought stress conditions. The reasons for this may be (1) early drought stress decreases tiller number, and (2) water deficit at grain filling induces carbon mobilization from tillers to the main stem ear [9,47]; therefore, the limited photosynthetic products are almost enough to ensure a good performance for grains of the main stem ear. Furthermore, grain size is greatly decreased by terminal drought [48]. TGW were drastically lower compared to materials not sprayed with desiccant (Fig. 2A, S3 Table).

### Genetic basis of the relationship between stem water-soluble carbohydrates in lower internodes and TGW under various drought stress conditions

Under various drought stress conditions, stem water-soluble carbohydrates are destined to have an inseparable relationship with drought stress, and are recognized as an important source for grain filling when current photosynthesis is inhibited by drought stress. It was also reported that accumulation and remobilization efficiencies of stem water-soluble carbohydrates differed between internodes, and that each internode responds differently to drought [49]. We found that the stem water-soluble carbohydrate in lower internodes were higher than those in peduncles (Fig. 1). Under diverse drought stress conditions, stem water-soluble carbohydrates in lower internodes had extremely significant correlations with TGW, especially at flowering under well-watered conditions and at the mid-grain filling under drought stress (Table 1). Thus, lower internodes should have sufficient length to store enough stem water-soluble carbohydrates and become a major source during the grain filling period [49].

### Pyramiding favorable alleles for stem water-soluble carbohydrates are effective for high and stable yield under various water conditions

Stem water-soluble carbohydrate in drought tolerant cultivars were observed to be higher than that in sensitive genotypes, both under control and stress conditions [13]. Among the 16 favorable alleles for stem water-soluble carbohydrates, five loci individually contributed to significantly higher 1000-grain weight (Table 2, Fig. 5). Pyramiding of favorable alleles for accumulation efficiency led to higher stem water-soluble carbohydrates and higher 1000-grain weight (Table 3). Because stem water-soluble carbohydrate and TGW are quantitative traits, individual loci among the numerous candidate genes are usually powerless to reveal the genetic basis and molecular relationships that underpin such complex traits. Perhaps this is the reason why only five of the 16 associated loci showed significant relationships between stem water-soluble carbohydrates and TGW. Our results indicated that 1) stem water-soluble carbohydrates can make a positive contribution to 1000-grain weight under variable water conditions; 2) pyramiding target favorable alleles is not only effective for obtaining genotypes with higher stem water-soluble carbohydrates, but also is effective for enhancing 1000-grain weight under drought conditions; and 3) the molecular relationships between stem water-soluble carbohydrates and 1000-grain weight are so complex that most of the single marker loci do not make measurable contributions. Our previous research identified seven novel favorable WSC alleles which exhibited positive individual contributions to 1000-grain weight, and were verified under 16 environments, including drought and heat stresses [50]. Among them, *Xgwm358–5D* (162 bp) is a common favorable WSC allele identified in the present research.

During the past 50 years, consistent gains in grain yield were made by empirical breeding. Stem water-soluble carbohydrates, as an important drought-tolerant related trait, was selected incidentally (Fig. 6). Moreover, trait-based selection inevitably pyramided some of the 16 favorable alleles identified in our research (Fig. 7). In the absence of epistasis, it should be possible to generate genotypes with higher numbers of favorable alleles and hence with the capability of higher WSC storage [20]. Scientists consider that significant increases in stem water-soluble carbohydrates have contributed to the recent genetic gains in grain yield in wheat [15,51]. High stem water-soluble carbohydrates has already been suggested as a criterion for wheat breeding under drought stress. With marker-assisted selection, accumulation of favorable alleles for water-soluble carbohydrates should play an important role in future wheat breeding programs.

## Supporting Information

S1 Table The 262 accessions and their origins. (XLSX)

S2 Table Statistical data for WSC in different internodes under various water conditions. (XLSX)

S3 Table Statistical data for 1000-grain weight (TGW) under various water conditions. (XLSX)

S4 Table Thirty-three loci significantly (*P* <0.01) associated with WSC. (XLSX)

S5 Table SSR markers significantly (*P* <0.01) associated with TGW. (XLSX)

S6 Table Statistical data for WSC and TGW under four water regimes. (XLSX)
